# Human odour thresholds are tuned to atmospheric chemical lifetimes

**DOI:** 10.1098/rstb.2019.0274

**Published:** 2020-04-20

**Authors:** Jonathan Williams, Akima Ringsdorf

**Affiliations:** Max Planck Institute for Chemistry, Hahn-Meitner Weg 1, 55128 Mainz, Germany

**Keywords:** odour threshold, atmospheric lifetime, volatile organic compounds (VOCs), olfaction, evolution

## Abstract

In this study, the odour thresholds (OT) and atmospheric lifetimes (AL) were compared for a suite of volatile organic compounds. It was found that odour threshold, as determined by the triangle bag method, correlated surprisingly well with atmospheric lifetime for a given chemical family. Molecules with short atmospheric lifetimes with respect to the primary atmospheric oxidant OH tend to be more sensitively detected by the human nose. Overall the correlation of odour threshold with atmospheric lifetime was better than with mass and vapour pressure. Several outliers from the correlations for particular chemical families were examined in detail. For example, diacetyl was an outlier in the ketone dataset that fitted the trend when its more important photolysis lifetime was included; and similarly, the relatively low odour threshold of carbonyl sulfide (OCS) was interpreted in terms of uptake by vegetation. The OT/AL relationship suggests that OH rate constants can be used as a first-order estimate for odour thresholds (and *vice versa*). We speculate that the nose's high sensitivity to chemicals that are reactive in the air is likely an evolved rather than a learned condition. This is based on the lack of dependence on ozone in the aliphatics, that the anthropogenically emitted aromatic compounds had the worst correlation, and that OCS had a much lower than predicted OT. Finally, we use the OT/AL relationships derived to predict odour thresholds and rate constants that have not yet been determined in order to provide a test to this hypothesis.

This article is part of the Theo Murphy meeting issue ‘Olfactory communication in humans’.

## 1. Introduction

Our sense of smell enables us to scan our environment for chemical signals. The chemical senses of smell and taste were the first to develop when life began, and even simple single-celled organisms are able to detect chemicals around them and react accordingly. Such signals may indicate an opportunity, such as a food source, or danger, such as poison. Therefore, a good sense of smell will be an evolutionary advantage for any organism. Some 200 000 years ago, our early human ancestors will have used their sense of smell to hunt, to differentiate nutritious from spoiled food, and to flee fire and predators. Nowadays, modern human beings increasingly rely on vision, with the result that we are losing active olfactory genes at a greater rate than all other primates [1,2]. It is interesting to speculate whether vestiges of the evolutionary development of our sense of smell can be ascertained today. The fact that a newborn baby uses smell to turn towards its mother's breast suggests that there is an inbuilt genetic coding for chemical detection [3]. On the other hand, the sense of smell is strongly linked to the neurobiology of memory, suggesting past personal experience is also an important factor in smell perception [4]. Finally, in the instant of smelling, our olfactory system reacts rapidly, desensitizing us to strong or persistent odours so as to retain detection capability [5]. The sense of smell is undoubtedly complex, and not yet fully understood.

**Figure 1.**
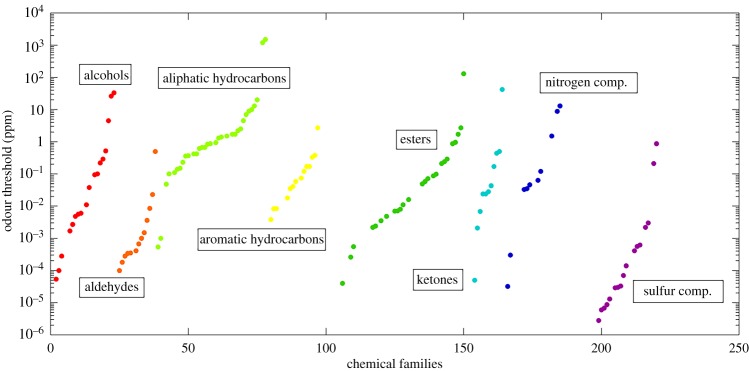
Odour thresholds from Nagata [14], plotted in chemical families, with each dot representing an individual chemical species. A lower odour threshold means it is detected at lower concentration. (Online version in colour.)

When human beings inhale, ambient air is drawn into the nasal cavity and brought into contact with the olfactory epithelium. Here trace components of the air interact with mucus-coated receptor cells. These transmit odour signals through the base of the skull to the olfactory bulb, an organ that is directly connected to the regions of the brain responsible for memory and emotion, the hippocampus and the amygdala. Each receptor detects more than one odour and each odour activates more than one receptor [6]. The overall sensation of odour results from inhaled volatile chemical compounds interacting with the olfactory area and registering in the brain. The smell experience of a particular chemical can be divided into two categories. The first is the perceived character of the smell, and the second is the intensity, which relates to how sensitively the molecule is detected [7]. In this study, we will focus on the innate sensitivity of the human nose to specific compounds as reflected by odour thresholds (OT). The human nose has a high sensitivity to many chemicals at low concentrations [8]. We can discern an immense range of odours, especially related to food, and with training can even track scents like dogs [9]. Human beings display a large variability in olfactory perception ability, dependent on factors such as genetics, gender, age, environment and health [10]. Although there is considerable variability between individuals, it is possible to determine a general detection capability of an average person to a specific chemical, and this is termed the odour threshold. This is effectively the concentration below which an average person cannot differentiate this particular molecule from clean air. Early attempts to determine odour thresholds were found to give very variable absolute results [11,12], albeit with similar relative values [13]. However, with better standardization and more rigorous methodology, results have proven reliable and reproducible. Recently, a comprehensive compilation of odour thresholds for 223 compounds was published by Yoshio Nagata from work done at the Japan Environmental Sanitation Center [14]. It was painstakingly acquired over 12 years using a panel of trained people, selected as their capability to detect smells represented the average for human beings (see Methods for details). These thresholds are shown in figure 1, grouped according to eight chemical families. The values span huge ranges, over six orders of magnitude, and are therefore plotted on a log scale. The most sensitively detected chemicals are the sulfur-containing species and the least the aliphatic alkanes. For the rest of this study, we will assume that the Nagata data represent the average odour threshold for human beings, and we will examine the distribution for indications that atmospheric chemistry has shaped or is shaping the sensitivity of the human nose.

**Figure 2.**
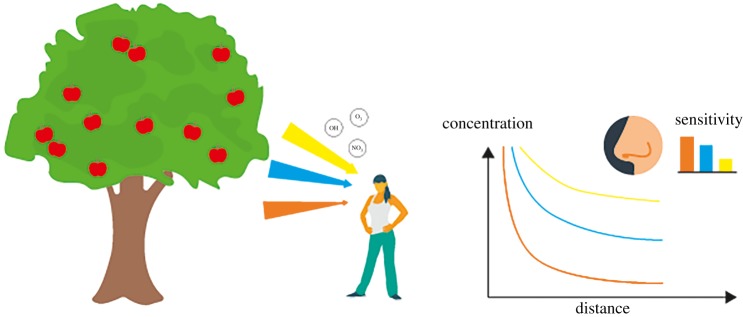
Schematic of the hypothesis that the sensitivity of the nose expressed as odour threshold will be inversely proportional to the rate of atmospheric removal. If ripe apples emit three odorous chemical species, with three different atmospheric lifetimes, then the nose will be most sensitive to the fastest-reacting species (orange).

The proposed connection between atmospheric chemistry and the sense of smell is indirect, and requires some explanation. When odorous molecules are released to the outside air (from a ripening fruit perhaps), their initial concentration will diminish with time and distance from the source as a result of mixing with cleaner surrounding air, and by photochemical oxidation. The process of oxidation transforms the chemical structure and therefore the odour of a molecule. The rate of chemical oxidation for a molecule is dependent on its structure, and odorous volatile organic compounds typically have chemical lifetimes of seconds to hours in ambient air [15]. Therefore, a scent concentration gradient will develop between the source (e.g. the fruit) and the seeker, owing to both mixing and chemistry. Short-lived species will have steep gradients over short distances and would, therefore, be useful for finding a nearby fruit in dense vegetation. Longer-lived species can signal the presence of fruit at a greater distance, as the odour will be carried further before the odour threshold is reached. However, long-lived species will exhibit weak scent gradients unsuitable for locating the fruit at close range. If the nose is tuned or has adapted through evolution to the atmospheric removal rates of the various odorous compounds then we may expect a relationship between the relative odour thresholds and the chemical removal rates—namely that short-lived molecules are detected more sensitively. This concept is shown schematically in figure 2.

The most important oxidant in the atmosphere is the hydroxyl radical (OH). These radicals act as chemical cleaning agents, reacting away toxic gases such as carbon monoxide (CO), and slowing climate warming by removing greenhouse gases like methane (CH_4_). Without OH, the air would be unbreathable to humans. The OH radical species also principally determines the lifetime of odorous gases in the atmosphere. The radicals form when ozone photolyses in sunlight to generate oxygen atoms (O^1^D), which subsequently react with water:

1.1 $${\text{O}}_{3}+\text{light}\to {\text{O}}^{1}\text{D + }{\text{H}}_{2}\text{O}\to \text{2OH}.$$

The OH radical can react with an odorous molecule either by abstracting a hydrogen atom and making water in the process, or by adding to a chemical double bond. In both cases, a chemical oxidation sequence is initiated that breaks down the molecule, ultimately to CO_2_ and H_2_O [15]. Since these reactions are important to the study of atmospheric chemistry, the rate of reaction of many molecules with OH has been determined in the laboratory under controlled conditions of temperature and pressure. If the rate of reaction with OH has been determined, it can be combined with a typical OH radical concentration (e.g. 1 × 10^6^ molecules cm^−3^) to generate an atmospheric lifetime (AL):

1.2 $$\text{AL}=\frac{1}{k[\text{OH}]},$$

where *k* is the rate constant of the reaction, and [OH] the concentration of OH radicals. The rate constants of many molecules with OH have been published as large compendiums such as the NIST kinetic database [16], and the IUPAC kinetic database [17]. Here we combine the latest odour threshold data from Nagata [14] with atmospheric lifetimes calculated from OH rate constants available in the literature.

## 2. Methods

### (a) The triangle odour bag method for determining odour thresholds

The determination of the human odour threshold has been performed with the triangle odour bag method developed in Japan. Between 1976 and 1988, Nagata conducted a long-term experiment applying this method to measure the odour threshold of 233 compounds which are used in this study [14].

For the triangle odour bag method as conducted by Nagata [14], trained panelists between 20 and 50 years old, who passed the screening test, were chosen to represent the standard human olfactory function. Four out of the six participants on the panel took part in the experiment over the whole period of 12 years. Each participant was tasked with distinguishing between odorous and odour-free air, inhaled through nose cones from 3 l polyester bags. For the triangle bag method, each panelist gets three bags, of which two contain odour-free air and one contains the odorant at a certain concentration. If the odorous bag was correctly identified, the concentration was sequentially diluted, until the panelist was not able to detect the odorous bag among the three choices any more. In the end, a single odour threshold concentration was determined for every compound, by excluding the minimum and maximum panelist value and then averaging over the remaining values. The obtained odour threshold is very similar to the detection threshold-reacting for the compound, since it is determined against odour-free air.

The concentrations of the odorants in the different bags are determined via the amount of the injected odour, referred to as primary odour and the dilution factor. The primary odours are taken from standard gas bottles or liquid standards. All gas samples are kept for a minimum of 2 h before use for the odour to diffuse [18]. The background gas used for the dilution was always nitrogen. Determining the concentration of the injected primary odour required different instrumentation depending on the compound. Ammonia was measured with the indophenol method, skatole and indole were analysed with a gas chromatograph mass spectrometer and for all other compounds a gas chromatograph with a flame ionization detector (FID), flame photometric detector (FPD) or flame thermionic detector (FTD) was used, calibrated with a standard pressurized gas.

The reproducibility of the results obtained with the described procedure was tested by repeating measurements of 25 out of the 233 compounds within the 12 years of experiment. The ratio of the highest to the lowest threshold was in the range of 1.2–5.2. The values were further validated by comparing them with measurements in an odourless chamber and with a triangle odour bag method experiment conducted with untrained panelists. The average odour thresholds and their dispersion do not differ significantly. An inter-laboratory comparison from 1985 with five participating laboratories showed that the results are 0.6–1.3 times the average value. This as well as an inter-laboratory experiment from 2002 indicate that the odour threshold by Nagata represent the average with relatively small variation, indicating that the OT measurements are robust.

### (b) OH rate determinations

The rate expression for a reaction is the equation describing the dependence of the rate on the concentration of reactants. The rate constant (*k*) is the constant of proportionality in the expression, relating rate of reaction to the concentrations of reactants or products. For the second-order reaction of OH radicals with an odorant molecule with abstractable hydrogen (RH), the rate expression is

rate=k[OH]×[RH].

The lifetime of RH with respect to OH is therefore given by equation (1.2). For the comparisons with odour threshold shown here we use [OH] = 1 × 10^6^ molecules cm^−3^. Although OH varies with season and altitude in the atmosphere the aforementioned value is typical for the Earth's surface by day at midlatitudes in summer [19].

The OH rate constant (*k*) for a reaction (e.g. OH + RH) is determined in the laboratory. Typically, the loss of small amounts of OH is followed in the presence of a great excess of RH. Alternatively, the rate constant may be determined relative to another well-established rate by reacting both odour molecules with OH together. The molecular physical properties that determine the OH rate constant include bond strength, number of bonds, electron density and energy barriers to intramolecular rearrangement. The rate constants for atmospheric molecules typically range from 1 × 10^−10^ to 1 × 10^−13^ cm^3^ molecule^−1^ s^−1^. The rate constants used here are taken from the NIST kinetic database, [16] or the IUPAC kinetic database [17]. The original reference for the exact rate used is given in the electronic supplementary material, table S1.

## 3. Results

### (a) Odour threshold correlations

**Figure 3.**
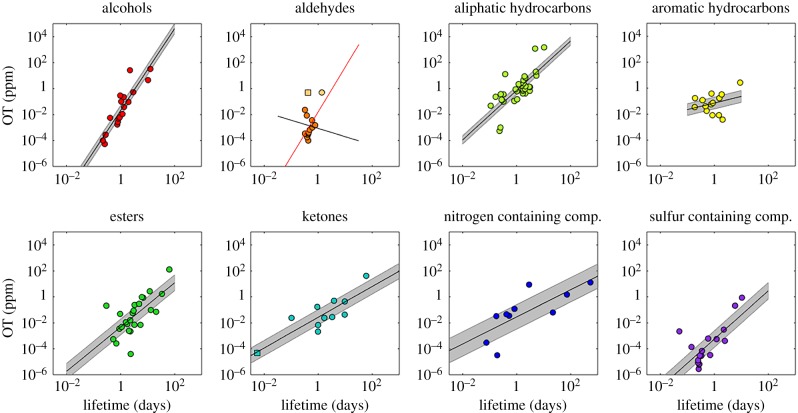
The odour threshold data from Nagata [14] are plotted against the atmospheric lifetimes of the species with respect to the OH radical. Shaded areas represent 95% confidence regions. The two square markers denote the compounds affected by photolysis: diacetyl in the ketone plot, and formaldehyde in the aldehyde plot. The two alternative fits for aldehydes, based on two formaldehyde lifetimes (uppermost pale yellow square and circle), are discussed in §3b 'Outliers'. (Online version in colour.)

In figure 3, we present odour threshold (OT) data plotted against atmospheric lifetime (AL) for eight chemical families (alcohols, aldehydes, aliphatic hydrocarbons, aromatic hydrocarbons, esters, ketones, nitrogen-containing compounds and sulfur-containing compounds). Since both odour thresholds and lifetimes span 3–6 orders of magnitude, the data are presented as log–log plots. A striking correlation is immediately apparent, whereby shorter-lived compounds have lower odour thresholds. These short-lived molecules are also the larger, higher mass molecules in the series, which generally have more abstractable hydrogen atoms available for reaction, and therefore react faster with OH. Interestingly, the correlation coefficient of the odour thresholds with OH lifetime is in all families better than that with mass (table 1). In the original paper by Nagata [14], the odour thresholds were plotted as a function of mass, and although there is a general tendency in the data for molecules with larger masses to have lower odour threshold, there is greater scatter than with atmospheric lifetime.

**Table 1.** Shows the Pearson's correlation of the original data for odour threshold against mass, vapour pressure (VP) and lifetime. The coefficients for the linear relationship of the log-transformed data and the associated *r*^2^ values are also given. HC, hydorcarbons.

family	Pearson correlation			linear regression	logplot coeff. of determination *r*^2^
	with mass	with VP	with lifetime		
alcohols	−0.5923	0.8285	0.6735	3.1584*x* − 1.7147	0.8028
aldehydes^a^	−0.4851	−0.193	−0.1220	−0.6334*x* − 3.0717	0.0046
aldehydes^b^	−0.4851	−0.193	0.8848	3.4439*x* − 1.7864	0.3365
aliphatic HC	−0.3591	0.5459	0.7473	1.9051*x* − 0.1496	0.53
aromatic HC	−0.7710	0.974	0.9566	0.5096*x* − 1.1416	0.0859
esters	−0.4537	0.8709	0.8455	1.7058*x* − 2.3207	0.4521
ketones	−0.6773	0.9203	0.9814	1.1845*x* − 1.5647	0.7157
nitrogen comp.	−0.1526	−0.2412	0.8025	1.0442*x* − 1.5353	0.5606
sulfur comp.	−0.1893	0.0974	0.9349	1.8615*x* − 3.2483	0.5115

^a^Aldehydes data pertaining to combined photolysis and OH lifetime of formaldehyde.

^b^Aldehydes data as regards only formaldehyde decomposition via OH for comparison.

Comparing the *r*^2^-values for the log plots shown in figure 3, the highest coefficients are observed for alcohols and the lowest for the benzenoid (aromatic) hydrocarbons. Direct emissions of alcohols to the atmosphere are predominately biogenic (i.e. from terrestrial vegetation, [20]) whereas aromatic compound emissions are primarily associated with fossil fuel processing and use. Interestingly, the alcohols, aldehydes, esters and ketones are all associated with food (e.g. fruit) while nitrogen and sulfur compounds are associated with urinary waste products and fire, respectively. Thus, it can be speculated that the relationship between OT and AL may have developed over evolutionary time through the ability to find food or to avoid danger.

Within the aliphatic chemical group correlation, there are both alkanes and alkenes. The alkenes contain double bonds which can react with ozone in addition to OH. Where ozone levels are high, this can be an additional loss process. We therefore compared the OT/AL relationship for OH only and for both OH and ozone. The inclusion of ozone in the atmospheric lifetime however, did not significantly improve the correlation.

### (b) Outliers

Within the plots shown in figure 3, most of the data form a reasonably clear correlation; however, some datapoints lie obviously outside the trend. We now examine these outliers in greater detail within the context of the hypothesis that the OT/AL correlation developed as an evolutionary process. It should be noted that in the following discussion we assume that both the odour threshold and rate measurement data are correct and do not discuss possible measurement error.

In the initial plot of all OTs and ALs for the family of ketones, a single outlier became immediately apparent. This was the species diacetyl, which has an OH lifetime in the atmosphere of 47 days. This compound showed an anomalously low odour threshold for such a long atmospheric lifetime. However, unlike almost all other species in this odour threshold database, diacetyl photolyses rapidly in sunlight (lifetime approx. 8 min), so its atmospheric lifetime is not determined by OH. If the faster photolysis lifetime is used, the datapoint for diacetyl moves close to the correlation fit, supporting the concept that odour threshold and atmospheric lifetime are linked.

Photolysis does not play a significant role in the atmospheric removal of the remaining species in the odour threshold dataset, with the exception of formaldehyde, which will be discussed in detail below. Chemical families such as the alkanes, alcohols and alkenes do not absorb light significantly at the photolysis-relevant actinic wavelengths, while for other species such as acetone the photolysis lifetime at the surface is negligible owing to the pressure dependence of the quantum yield. Wet and dry deposition may also play a minor role, particularly in forested environments; however, these loss rates are currently too uncertain and variable to be considered in detail with this study. We therefore confine our analysis to OH radicals, which are the main loss process for VOCs in the atmosphere, and two additional photolysis loss rates (i.e. diacetyl and formaldehyde). Formaldehyde photolysis is highly dependent on time of day and latitude. The full day average photolysis lifetime at midlatitudes has been estimated to be 15 h [21]; however, instantaneous values can be less than an hour and variable through the day [22]. It is therefore difficult to determine an appropriate value for this study, and therefore we show a range of lifetimes for formaldehyde in figure 3 (from OH only to OH and photolysis). Interestingly, when only the OH lifetime is used, formaldehyde forms a good correlation with the other aldehydes, indeed better than that obtained with mass. Shorter formaldehyde lifetimes (with photolysis included) cause a rapid degradation of the correlation coefficient as the other aldehydes in the dataset are unfortunately all of similar lifetime. If the OT/AL relationship for aldehydes could be better defined with longer-lived species one could deduce the apparent lifetime of formaldehyde from the odour threshold. This could be perhaps interpreted in terms of the latitudinal regions in which the human sense of smell evolved (or in this case the Japanese group tested for this dataset). However, given the large variations in formaldehyde photolysis rates with forest cover, altitude, latitude and time of day this would be extremely uncertain. For completeness, in figure 3 and for the the coefficients in table 1 the formaldehyde atmospheric lifetime includes both relationships: OH alone (33 h) and combined OH and photolysis (10 h).

For the sulfur compound family, DMDS (dimethyl disulfide) has a higher odour threshold than would be expected from the OT/AL correlation. Why DMDS is so far removed from the line is not clear. One possibility is that DMDS is usually emitted together with the more odorous DMS (dimethyl sulfide), typically as approximately 10% of the DMS signal [23]. Another possible explanation is that DMDS is emitted from spoiled or rotting meat [24]. Although determining whether meat is safe to eat would be an evolutionary advantage, such assessments are made by smelling at close quarters, where air chemistry plays little role. A second sulfur compound, carbonyl sulfide (OCS), was excluded from the plot on the basis of its very long lifetime (4–7 years). This was considered incompatible with the hypothesis considered. Interestingly, the compound OCS has an anomalously low odour threshold given this long atmospheric lifetime, at least under present-day conditions. The main loss process of OCS from the atmosphere is not due to OH, but instead irreversible uptake by plants during photosynthesis. If this odour threshold has been determined evolutionarily, it suggests that the lifetime of OCS was much shorter in the past. Indeed, 10 000 years ago, forest cover was significantly greater than today (by approx. a factor of 4 in Europe) and many early hominoids would have dwelt in the forest. Although admittedly speculative, this archaeological atmospheric chemistry interpretation of the OCS data does have at least the right tendency and thus is consistent with the evolutionary development of odour thresholds.

The family of aromatic hydrocarbons had the lowest correlation coefficients. Indeed four compounds in particular stand out, even apparently showing the opposite OT/AL trend. These are *n*-benzene, iso-propyl benzene, and ethyl toluene. Although several benzenoid (aromatic) compounds such as toluene are emitted from vegetation [25,26], the non-aligned species named above are primarily associated with anthropogenic oil- and gas-related emissions. Therefore, a possible explanation of the poor linear relationship with the aromatics is that they are too new for a selectivity to have evolved. Moreover, there is no evolutionary pressure to find a petrol station.

## 4. Discussion

By connecting the odour threshold (OT) and OH reaction rate datasets, we have revealed a strong correlation within chemical families for most of the species considered. Interestingly, the correlation between odour threshold and atmospheric lifetimes (AL) for the chemical families examined (e.g. alcohols, esters, ketones) was stronger than the correlation with mass, which had been previously examined. Since mass can be a proxy for volatility or diffusion across the olfactory epithelium, both potentially important attributes within the olfactory detection system, the correlation with atmospheric lifetime is remarkable. The odour thresholds (OTs) can also be correlated against other potentially relevant physical properties. For completeness, we also examined the relationship with vapour pressure and found that while vapour pressure correlations were comparable with those found for atmospheric lifetimes (i.e. better than with mass) for some chemical families, the vapour pressure correlation broke down completely for the sulfur and the nitrogen-containing families. Therefore, the OT/AL relationship was better than OT/mass for all chemical families except the aldehydes, which were uncertain owing to formaldehyde, and better than vapour pressure overall as it also accounted for the behaviour of nitrogen and sulfur compounds. This supports the importance of the chemical lifetime in olfaction and suggests that the human nose has adapted to detect short-lived odours in air most sensitively.

Several aspects of this dataset suggest that the OT/AL relationship has developed over long time periods through evolutionary selection. Firstly, the emissions from foodstuffs (alcohols and aldehydes) are correlated much better than modern-day anthropogenic species (aromatic hydrocarbons). Secondly, the atmospheric lifetime of OCS, which is primarily determined by the extent of global vegetation, was much shorter than current estimates, consistent with the known much greater forest cover in the past. Thirdly, no improvement was observed in the odour threshold–atmospheric lifetime fit when ozone was also considered for the aliphatics including double-bonded compounds. This suggests ozone levels played little role in influencing the odour thresholds, as would have been the case over most of human history. Past ozone levels were much lower than today's as the emissions of the anthropogenic precursor nitrogen oxides (NO and NO_2_) were much lower than today. For this reason, ozone over pristine forests is significantly lower than in urban areas [27].

If the sensitivity of our sense of smell has developed evolutionarily, we may expect that it was an important sense for our ancestors. Indeed, several species of primates have been shown to be sensitive to food odours [28] and use chemical odours in foraging [29–33]. In experiments on wild and semi-wild New World primates, owl monkeys, one out of two emperor tamarin groups, found fruit-baited locations based on olfactory cues [34]. However, several other species of primates failed in this task, so that the importance of olfaction in primate foraging over longer distances remains unclear. Over shorter distances, olfaction does play a role, as noted in slender lorises foraging for invertebrates [35]. Generally, in the relatively dark and obstructed environment of a forest, the sense of smell would seem to be extremely useful in locating food sources and avoiding danger.

The potential connection between OT and AL highlighted in this work can be tested both by the odour threshold community and by atmospheric scientists. To test this relationship, we predict, based on the relationships shown here, seven new (unmeasured) odour thresholds and 12 new OH rate constants. To our knowledge, there are no data on the molecules we have selected and so this represents a good test of the hypothesis (table 2).

**Table 2.** A list of predicted atmospheric lifetimes (ALs) based on odour threshold (OT) relationships, and a list of predicted odour thresholds based on atmospheric lifetimes.

	OT (ppm)	predicted lifetime (days)	
*n*-nonanol	9.00 × 10^−4^	0.379	
geosmin	6.50 × 10^−6^	0.080	
tetralin	9.30 × 10^−3^	0.103	
3-methylheptane	1.50	1.482	
*n*-butylbenzene	8.50 × 10^−3^	0.015	
*m*-diethylbenzene	7.00 × 10^−2^	0.942	
ethyl *n*-valerate	1.10 × 10^−4^	0.110	
isopropyl ethanoate	1.60 × 10^−1^	7.832	
isopropylamine	2.50 × 10^−2^	0.863	
*n*-butylamine	1.70 × 10^−1^	5.412	
*n*-hexylmercaptan	1.50 × 10^−5^	0.142	
allyl sulfide	2.20 × 10^−4^	0.603	
	predicted OT (ppm)	lifetime (days)	source
allyl alcohol	2.16 × 10^−4^	0.241	Le Person *et al.* [36]
cyclobutane	1.95 × 10^1^	5.702	Atkinson [37]
methoxybenzene	6.35 × 10^−2^	0.777	Tomohiro & Toshiro [38]
*n*-pentyl acetate	1.04 × 10^−2^	1.577	El Boudali *et al*. [39]
3-pentanone	2.39 × 10^−1^	6.256	Atkinson *et al*. [40]
methylamine	1.49 × 10^−2^	0.526	Atkinson [41]
ethyl mercaptan	4.37 × 10^−5^	0.253	Atkinson [41]

Given the requisite equipment, the OH rate constant can be determined absolutely by physical measurement reasonably quickly (approximately hours). By contrast, the OT determination requires multiple people to assess an odour over many dilutions, and the result is a biologically determined average perception. Therefore, it may be easier to generate useful new odour thresholds by measuring OH rates. Based on the correlations shown in this study, we may expect the OH rate constant-derived OT to be a good first-order estimate, provided that the molecule in question is primarily removed from the atmosphere by OH (rather than by deposition or photolysis).

Given the focus of this special issue, it is interesting to discuss the relationships presented here in the context of human olfactory signal broadcasting and perception. From the data shown here, it is tempting to speculate that human chemical emissions related to signalling at close quarters (e.g. related to procreation) will be short lived, whereas signals warning others away should be somewhat longer lived, giving a wider detectable range. The only mammalian pheromone identified to date is 2-methylbut-2-enal, which is found in rabbit milk [42], has an OH rate constant of 4.67 × 10^−11^ molecules cm^−3^ s^−1^, corresponding to an OH-determined atmospheric lifetime of 0.24 days. On the scale of odour thresholds, this is moderate to low. Ripening fruits emit multiple VOC cues with a wide span of atmospheric lifetimes and hence odour thresholds. This potentially allows experienced human foragers to gauge their proximity to a food source since as the fruit is approached more short-lived (and sensitively detected) species will add to the scent. Since primates consume the fruit and disperse the seeds some degree of coevolution of the signals may be expected. Furthermore, if the nose is evolutionarily tuned, it is tempting to think about odour thresholds as indicators of the relative importance of certain foodstuffs or dangers to human survival. Certainly, the high sensitivity to sulfur compounds (figure 1) suggests fire to be an evolutionary pressure. Among the extremely well-correlated alcohols, geosmin is the most sensitively detected followed by *p*-cresol. Geosmin is an indicator of bacteria-contaminated water, while *p*-cresol is excreted in human faeces and urine. It is possible that these molecules served as markers of human presence and pathogens, similar to skatole, the most sensitively detected nitrogen-containing compound. Finally, the most sensitively detected ester is ethyl isobutyrate, which has a fruity and sweet odour and therefore is likely to be associated with food gathering.

In summary, we show and discuss the correlation between odour threshold and atmospheric lifetime. The correlations and outliers are generally consistent with an evolutionary tuning of the human nose sensitivity to molecules with short atmospheric lifetimes. Independent support for the evolutionary tuning of our sense of smell based on RNA sequencing of mammals has been very recently published [43]. The relationships between OT and AL have been exploited to make predictions of both OTs and ALs for previously unmeasured compounds. Since determining OT and AL requires specialized equipment, these relationships represent a cost-effective way of estimating these parameters. With larger datasets (as possessed by fragrance companies), it may be possible to gain further insights into our evolutionary past, opening an entirely new field of air chemical-based archaeology. Finally, we note that modern humans spend over 90% of their life indoors, where OH is low [44]. This may well impact the pattern of odour thresholds in the future, and in this context, it would be interesting to test the OT/AL relationships for both hunter–gatherers and city dwellers.

## Supplementary Material

Table S1

## Data accessibility

The data are provided as electronic supplementary material.

## References

[1] Gilad Y, Wiebe V, Przeworski M, Lancet D, Pääbo S 2004 Loss of olfactory receptor genes coincides with the acquisition of full trichromatic vision in primates. PLoS Biol. 5, e148 (10.1371/journal.pbio.0020005) PMC314465 14737185

[2] Sarafoleanu C, Mella C, Georgescu M, Perederco C 2009 The importance of the olfactory sense in the human behavior and evolution. J. Med. Life 2, 196–198. 20108540 PMC3018978

[3] Doucet S, Soussignan R, Sagot P, Schaal B 2009 The secretion of areolar (Montgomery's) glands from lactating women elicits selective, unconditional responses in neonates. PLoS ONE 4, e7579 (10.1371/journal.pone.0007579) 19851461 PMC2761488

[4] Wilson DA, Best AR, Sullivan RM 2004 Plasticity in the olfactory system: lessons for the neurobiology of memory. Neuroscientist 10, 513–524. (10.1177/1073858404267048) 15534037 PMC1868530

[5] Dalton P 2000 Psychophysical and behavioral characteristics of olfactory adaptation. Chem. Senses 25, 487–492. (10.1093/chemse/25.4.487) 10944515

[6] Buck L, Axel R 1991 A novel multigene family may encode odorant receptors: a molecular basis for odor recognition. Cell 65, 175–187. (10.1016/0092-8674(91)90418-X) 1840504

[7] Keller A, Vosshall LB 2016 Olfactory perception of chemically diverse molecules. BMC Neurosci. 17, 55 (10.1186/s12868-016-0287-2) 27502425 PMC4977894

[8] Shepherd GM 2004 The human sense of smell: are we better than we think? PLoS Biol. 2, e146 (10.1371/journal.pbio.0020146) 15138509 PMC406401

[9] Porter J, Craven B, Khan RM, Chang SJ, Kang I, Judkewitz B, Volpe J, Settles G, Sobel N 2007 Mechanisms of scent-tracking in humans. Nat. Neurosci. 10, 27–29. (10.1038/nn1819) 17173046

[10] Stevens JC, Cain WS, Burke RJ 1988 Variability of odour thresholds. Chem. Senses 13, 643–653. (10.1093/chemse/13.4.643)

[11] Amoore JE 1980 Properties of olfactory system. In Institute of Gas Technology Symposium on Odorization (eds Suchomel FH, Weatherly JW), pp. 31–35. Chicago, IL: Institute of Gas Technology.

[12] Yoshida M 1984 Correlational analysis of detection threshold data for ‘standard test’ odors. Bull. Fac. Sci. Eng. Chuo Univ. 27, 343–353.

[13] Punter PH 1983 Measurement of human odour thresholds for several groups of structurally related compounds. Chem. Senses 7, 215–235. (10.1093/chemse/7.3-4.215)

[14] Nagata Y 2003 Measurement of odor threshold by triangle odor bag method. In Odor measurement review, pp. 118–127. Tokyo, Japan: Office of Odor, Noise and Vibration, Environmental Management Bureau, Ministry of the Environment, Government of Japan.

[15] Williams J 2004 Organic trace gases: an overview. Environ. Chem. 1, 125–136. (10.1071/EN04057)

[16] Manion JA et al 2015 *NIST chemical kinetics database, NIST standard reference database 17, version 7.0 (Web version), release 1.6.8, data version 2015.09* Gaithersburg, MD: National Institute of Standards and Technology See http://kinetics.nist.gov/.

[17] Atkinson R et al. 2006 Evaluated kinetic and photochemical data for atmospheric chemistry: volume II – gas phase reactions of organic species. Atmos. Chem. Phys. 6, 3625–4055. (10.5194/acp-6-3625-2006)

[18] Iwasaki Y 2003 The history of odor measurement in Japan and triangle odor bag method. In Odor measurement review, pp. 37–47. Tokyo, Japan: Office of Odor, Noise and Vibration, Environmental Management Bureau, Ministry of the Environment, Government of Japan.

[19] Spivakovsky CM et al 2000 Three-dimensional climatological distribution of tropospheric OH: update and evaluation. J. Geophys. Res. 105, 8931–8980. (10.1029/1999JD901006)

[20] Guenther AB, Jiang X, Heald CL, Sakulyanontvittaya T, Duhl T, Emmons LK, Wang X 2012 The model of emissions of gases and aerosols from nature version 2.1 (MEGAN2.1): an extended and updated framework for modeling biogenic emissions. Geosci. Model Dev. 5, 1471–1492. (10.5194/gmd-5-1471-2012)

[21] Warneck P 2001 Chemistry of the natural atmosphere. New York, NY: Academic Press Inc.

[22] Lee Y-N et al. 1998 Atmospheric chemistry and distribution of formaldehyde and several multioxygenated carbonyl compounds during the 1995 Nashville/Middle Tennessee Ozone Study. J. Geophys. Res. 103, D17 (10.1029/98JD01251)

[23] Williams J, Pöschl U, Crutzen PJ, Hansel A, Holzinger R, Warneke C, Lindinger W, Lelieveld J 2001 An atmospheric chemistry interpretation of mass scans obtained from a proton transfer mass spectrometer flown over the tropical rainforest of Surinam. J. Atmos. Chem. 38, 133–166. (10.1023/A:1006322701523)

[24] Mayr D, Margesin R, Klingsbichel E, Hartungen E, Jenewein D, Schinner F, Märk TD 2003 Rapid detection of meat spoilage by measuring volatile organic compounds by using proton transfer reaction mass spectrometry. Appl. Environ. Microbiol. 69, 4697–4705. (10.1128/AEM.69.8.4697-4705.2003) 12902260 PMC169070

[25] Heiden AC, Kobel K, Komenda M, Koppmann R, Shao M, Wildt J 1999 Toluene emissions from plants. Geophys. Res. Lett. 26, 1283–1286. (10.1029/1999GL900220)

[26] Misztal PK et al 2015 Atmospheric benzenoid emissions from plants rival those from fossil fuels. Scient. Rep. 5, 12064 (10.1038/srep12064) PMC4499884 26165168

[27] Williams J et al 2016 Opposite OH reactivity and ozone cycles in the Amazon rainforest and megacity Beijing: subversion of biospheric oxidant control by anthropogenic emissions. Atmos. Environ. 125A, 112–118. (10.1016/j.atmosenv.2015.11.007)

[28] Laksa M, Seibt A, Weber A 2000 Microsmatic primates revisited: olfactory sensitivity in the squirrel monkey. Chem. Senses 25, 47–53. (10.1093/chemse/25.1.47) 10667993

[29] Vickers NJ 2000 Mechanisms of animal navigation in odor plumes. Biol. Bull. 198, 203–212. (10.2307/1542524) 10786941

[30] Dominy NJ, Lucas PW, Osorio D, Yamashita N 2001 The sensory ecology of primate food selection. Evol. Anthropol. 10, 171–181. (10.1002/evan.1031) 36519416

[31] Bicca-Marques JC, Garber PA 2004 Use of spatial, visual and olfactory information during foraging in wild nocturnal and diurnal anthropoids: a field experiment comparing *Actus, Callicebus, and Saguinus*. Am. J. Primatol. 62, 171–187. (10.1002/ajp.20014) 15027091

[32] Nevo O, Garri RO, Hernandez Salazar LT, Schulz S, Heymann EW, Ayasse M, Laska M 2015 Chemical recognition of fruit ripeness in spider monkeys (*Ateles geoffroyi*). Scient. Rep. 5, 14895 (10.1038/srep14895) PMC4594300 26440380

[33] Nevo O, Heymann EW, Schulz S, Ayasse M 2016 Fruit odor as a ripeness signal for seed-dispersing primates? A case study on four Neotropical plant species. J. Chem. Ecol. 42, 323–328. (10.1007/s10886-016-0687-x) 27039380 PMC4869761

[34] Bolen RH, Green SM 1997 Use of olfactory cues in foraging by owl monkeys and capuchin monkeys. J. Comp. Psychol. 111, 152–158. (10.1037/0735-7036.111.2.152) 9170280

[35] Nekaris KAI 2005 Foraging behaviour of the slender loris (*Loris lydekkerianus lydekkerianus*): implications for theories of primate origins. J. Hum. Evol. 49, 289–300. (10.1016/j.jhevol.2005.04.004) 15970312

[36] Le Person A, Solignac G, Oussar F, Daële V, Mellouki A, Winterhalter R, Moortgat GK 2009 Gas phase reaction of allyl alcohol (2-propen-1-ol) with OH radicals and ozone. Phys. Chem. Chem. Phys. 11, 7619–7628. (10.1039/b905776e) 19950501

[37] Atkinson R 2003 Kinetics of the gas-phase reactions of OH radicals with alkanes and cycloalkanes. Atmos. Chem. Phys. 3, 2233–2307. (10.5194/acp-3-2233-2003)

[38] Tomohiro O, Toshiro O 1985 A set of rate constants for the reactions of OH radicals with aromatic hydrocarbons. Bull. Chem. Soc. Jpn 58, 10 (10.1246/bcsj.58.3029)

[39] El Boudali A, Le Calvé S, Le Bras G, Mellouki A 1996 Kinetic studies of OH reactions with a series of acetates. J. Phys. Chem. 100, 12 364–12 368. (10.1021/jp9606218) 32223123

[40] Atkinson R, Aschmann SM, Carter WPL, Pitts JN Jr 1982 High-temperature measurements of the reactions of OH with a series of ketones: acetone, 2-butanone, 3-pentanone, and 2-pentanone. Int. J. Chem. Kinetics 14, 839–847. (10.1002/kin.550140804) 22607582

[41] Atkinson R 1986 Kinetics and mechanisms of the gas-phase reactions of the hydroxyl radical with organic compounds under atmospheric conditions. Chem. Rev. 86, 69–201. (10.1021/cr00071a004)

[42] Schaal B, Coureaud G, Langlois D, Giniès C, Sémon E, Perrier G 2003 Chemical and behavioural characterization of the rabbit mammary pheromone. Nature 424, 68–72. (10.1038/nature01739) 12840760

[43] Saraiva LR et al 2019 A transcriptomic atlas of mammalian olfactory mucosae reveals an evolutionary influence on food odor detection in humans. Sci. Adv. 5, eaax0396 (10.1126/sciadv.aax0396) 31392275 PMC6669018

[44] Klepeis NE, Nelson WC, Ott WR, Robinson JP, Tsang AM, Switzer P, Behar JV, Hern SC, Engelmann WH 2001 The National Human Activity Pattern Survey (NHAPS): a resource for assessing exposure to environmental pollutants. J. Exposure Anal. Environ. Epidemiol. 11, 231–252. (10.1038/sj.jea.7500165) 11477521

